# Macrophage signaling in HIV-1 infection

**DOI:** 10.1186/1742-4690-7-34

**Published:** 2010-04-09

**Authors:** Georges Herbein, Gabriel Gras, Kashif Aziz Khan, Wasim Abbas

**Affiliations:** 1 Department of Virology, UPRES 4266 Pathogens and Inflammation, IFR 133 INSERM, University of Franche-Comté, CHU Besançon, F-25030 Besançon, France; 2 CEA, Institute of Emerging Diseases and Innovative Therapies, Division of Immuno-Virology, Université Paris-Sud UMR E01, F-92265 Fontenay-aux Roses, France

## Abstract

The human immunodeficiency virus-1 (HIV-1) is a member of the lentivirus genus. The virus does not rely exclusively on the host cell machinery, but also on viral proteins that act as molecular switches during the viral life cycle which play significant functions in viral pathogenesis, notably by modulating cell signaling. The role of HIV-1 proteins (Nef, Tat, Vpr, and gp120) in modulating macrophage signaling has been recently unveiled. Accessory, regulatory, and structural HIV-1 proteins interact with signaling pathways in infected macrophages. In addition, exogenous Nef, Tat, Vpr, and gp120 proteins have been detected in the serum of HIV-1 infected patients. Possibly, these proteins are released by infected/apoptotic cells. Exogenous accessory regulatory HIV-1 proteins are able to enter macrophages and modulate cellular machineries including those that affect viral transcription. Furthermore HIV-1 proteins, e.g., gp120, may exert their effects by interacting with cell surface membrane receptors, especially chemokine co-receptors. By activating the signaling pathways such as NF-kappaB, MAP kinase (MAPK) and JAK/STAT, HIV-1 proteins promote viral replication by stimulating transcription from the long terminal repeat (LTR) in infected macrophages; they are also involved in macrophage-mediated bystander T cell apoptosis. The role of HIV-1 proteins in the modulation of macrophage signaling will be discussed in regard to the formation of viral reservoirs and macrophage-mediated T cell apoptosis during HIV-1 infection.

## Introduction

HIV-1 infection is characterized by sustained activation of the immune system. As macrophages, along with other cell types, are permissive to HIV-1 infection, they may be infected by the virus, resulting in signaling modulation [1]. Even uninfected macrophages may be activated by the soluble gp120 HIV-1 protein, or gp120 virion, via several signaling pathways. Additionally, soluble HIV-1 proteins such as Nef, Tat, and Vpr have been detected in serum of HIV-1 infected patients, possibly released by infected/apoptotic cells. Soluble exogenous HIV-1 proteins are able to enter macrophages and modulate both cellular machinery and viral transcription. Deciphering the signaling pathways involved in the activation of macrophages in HIV infection is critical to a better understanding of AIDS pathogenesis as this could lead to innovative therapeutic approaches.

## HIV-1 Proteins and Macrophage Signaling

### Nef

Nef is a 27-kDa myristylated protein which is expressed early in the virus life cycle. Nef down-regulates the cell surface expression of CD4, CD28, and MHC class I [2]. Nef also modulates several signaling pathways [3-8]. While Nef is not considered to be a secreted protein, exogenous Nef has been detected in the sera of AIDS patients and in cultures of HIV-1-infected cells [9]. There is increasing evidence of the ability of extracellular Nef to activate signaling pathways in uninfected cells [9-13]. Indeed, Nef is internalized by MDMs and dendritic cells, but not by T cells [14], when added to cell cultures [14-16]. Recently, Qiao *et al. *[11] reported that Nef was internalized in B cells *in vitro*, thereby suppressing CD40-dependent immunoglobulin class switching. The presence of Nef in the sera of HIV-infected patients at concentrations ranging from 1 to 10 ng/mL has also been described [9]. This concentration may be higher in the lymphonodal germinal centers where virion-trapping dendritic cells, as well as virion-infected CD4+ T cells and macrophages, are densely packed [17,18]. Infected cells may release Nef through a non-classical secretory pathway or after lysis. Following this, bystander cells may internalize Nef via endocytosis, pinocytosis or other yet-unknown mechanisms. Regarding intracellular signaling induced by Nef treatment of MDMs, it has been reported that Nef modulates the expression of a significant number of genes as early as 2 hours after treatment [19]. This suggested that a prompt transcriptional cell reprogramming induced by Nef leads to the synthesis and the release of pro-inflammatory cytokines/chemokines, which in turn, activate STAT1 and STAT3 signal transducers and transcription activators [20,21]. In line with these results, Nef treatment of MDMs was reported to induce rapid activation of IKK/NF-kB, MAPK and IRF-3 signalling pathways. Nef induces prompt phosphorylation of three MAPKs, i.e., ERK1/2, JNK, and p38 [13,22,23]. A Nef treatment as short as 15 minutes is able to induce p38 phosphorylation, most likely due to rapid recruitment and activation of p38 signaling upstream intermediates. Exogenously added Nef induces rapid phosphorylation of the transcription factor IRF-3, the main regulator of IFN-β gene expression [24-26]. It has also been shown to induce tyrosine phosphorylation of STAT2, well known to be induced by type I IFN signaling, at an early infection stage (8 to 16 h) [22].

**Figure 1:**
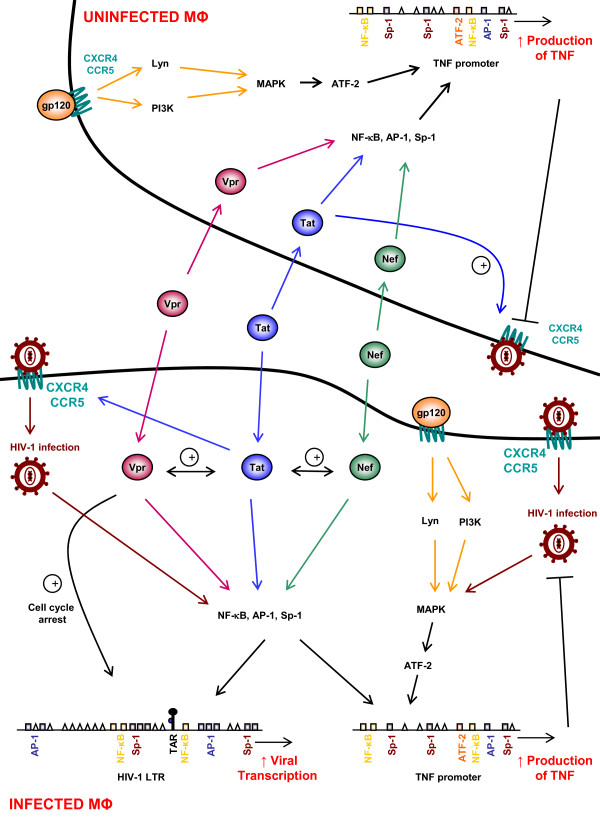
**HIV-1 proteins modulate signaling in the macrophage**. The HIV-1 proteins Nef, Tat, Vpr, and gp120 alter cell signaling pathways, both in infected and uninfected macrophages. The presence of exogenous Nef, Tat and Vpr has been reported in sera of AIDS patients, which have the ability to enter the cells. HIV-1 proteins activate multiple transcription factors in macrophages including NF-κB, Sp-1 and AP-1, which have binding sites in the long terminal repeat (LTR) of HIV-1. The induction of these factors results in increased viral production. Furthermore, the activation of these transcription factors enhances cytokine production by macrophages primarily involved in AIDS pathogenesis. TNF promoter is shown as a prototype containing binding sites of NF-κB, Sp-1, and AP-1. Exogenous Nef and Vpr may enhance Tat-mediated transcription in addition to their effect on transcription factors. Moreover, the viral glycoprotein gp120 activates MAPK in uninfected and infected cells, resulting in increased TNFα production through ATF-2 binding sites of its promoter. Tat also stimulates CXCR4/CCR5 surface co-receptor expression, thus enhancing viral entry in cells. Besides LTR activation through transcription factors, Vpr-induced cell cycle arrest facilitates LTR stimulation.

Macrophage activation and production of pro-inflammatory cytokines by Nef involves NF-κB activation, especially its p50/p50 homodimeric and p65/p50 heterodimeric forms. This event leads to sustained LTR activation [13,19,27]. The activation of NF-κB in macrophages treated with exogenously added Nef occurs as early as 2 hours after treatment [13,28]. NF-κB activation in primary macrophages treated with recombinant Nef is mediated via the canonical pathway, primarily involving IKKβ phosphorylation [28]. Furthermore, many of the transcripts induced in macrophages treated by Nef are encoded by genes regulated by κB-like responsive elements [19] (Figure 1). Therefore, there is evidence that exogenously added Nef plays a critical role in "hijacking" the NF-κB signaling pathway, most likely upstream of IKK, as observed after endogenous expression in macrophages [29]. This observation is in line with the role of Nef-mediated activation of NF-κB, which promotes HIV-1 replication via both direct and cytokine-mediated effects [13]. Thus, in monocyte-derived macrophages, recombinant Nef enhances the production of cytokines such as macrophage inflammatory protein-1 alpha (MIP1α), MIP1β, TNFα, IL-1β and IL-6 involved in the inflammatory response (Figure 1). Additionally, features observed in promonocytic cells and primary macrophages following exposure to recombinant Nef are very similar to those observed following TNFα treatment [30]. Both recombinant Nef and TNFα activate NF-κB, AP-1 and JNK. That recombinant Nef and TNFα activate these signaling pathways suggests the two events might modulate the cellular machinery in a similar way. Therefore, they may have the same effects on HIV-1 replication in mononuclear phagocytes [28]. Exogenous Nef may modulate intracellular signaling pathways downstream of the TNFα receptors (TNFRs), and thus mimic the effects of TNFα on primary macrophages [13].

### Tat

HIV-1 Tat is a virally encoded transactivating protein which plays a critical role in viral replication and is conserved in genomes of primate lentiviruses [31,32]. Tat is a HIV-1 protein reportedly detected in the sera of infected patients as well as in the media of infected cells [33]. This suggests that it might have a role both as endogenous modulator of cellular functions within infected cells and act on bystander cells. Tat activates monocytes, macrophages, and microglial cells.

#### Tat Action on monocytes, macrophages, and monocytic cell lines

The HIV-1 Tat protein is essential for efficient transcription of viral genes and for viral replication. It also regulates the expression of several cellular genes and interferes with intracellular signaling [34,35]. The mature protein has a variable size, ranging from 86 to 101 amino acids. It is organized in functional domains required for transactivation activity. The C-terminus contains an RDG motif which mediates cell adhesion and Tat binding to integrin receptors [36]. Specific Tat binding has been reported for at least three cell surface molecules including heparin sulfate, beta-integrin and chemokine receptors. Tat as well as peptides spanning its cysteine-rich region compete with cognate ligands to bind CXCR4, CCR2, and CCR3 chemokine receptors in primary human monocytes and PBMCs. Tat has also been reported to trigger Ca^2+ ^mobilization in macrophages in a concentration-dependent manner through CCR2 and CCR3 [37,38]. Moreover, Tat induces the expression of CCR3, CCR5 and CXCR4 in monocytes/macrophages in a concentration-dependent manner, possibly promoting HIV-1 infection [39]. Finally, Tat has been shown to serve as chemoattractant for monocytes, and pretreatment with Tat enhanced the monocyte invasive properties [40,41].

Functional consequences of Tat activation include TNFα release from macrophages, monocytes and THP-1 monocytic cell lines [42]. Tat-induced TNFα release was dependent on NF-κB activation and mediated through the activation of protein kinase A, phospholipase C (PLC) and protein tyrosine kinase pathways [43]. Transient [Ca^2+^]i release was observed in macrophages through IP3 receptor-regulated intracellular Ca^2+ ^stores [43]. This Tat-induced [Ca^2+^]i elevation was not dependent on extracellular Ca^2+ ^or caffeine-sensitive ryanodine receptor-regulated intracellular Ca^2+ ^stores but rather on the PLC, protein kinase C (PKC) and Gi/0 protein pathways. Tat-induced calcium signaling in macrophages leads to the production of pro-inflammatory cytokines and chemokines, possibly contributing to inflammation and HIV-1 neuropathogenesis.

Thus, Tat displays biological activities mimicking those mediated by TNFα [28]. HIV-1 Tat may induce the expression of TNFα and various cytokines, including IL-6, TNFβ and TGFβ as well as the expression of cytokine receptors such as the IL-4 receptor [44-49]. Like TNFα, Tat may activate NF-κB, AP-1 and MAPK, including c-Jun N-terminal kinase/stress-activated protein kinase (JNK/SAPK) [50]. Tat activates NF-κB, JNK, and AP-1, but not MEK [50]. These results suggest that HIV-1 Tat and TNFα act through different mechanisms and that HIV-1 Tat does not activate all of the kinases involved in TNFR signaling [51]. In short, like Nef, Tat mimics the effects of TNFα resulting in the enhancement of viral replication via activation of NF-κB, AP-1, JNK, and MAPK.

#### Action of Tat on microglia

Tat protein is actively produced and released in the central nervous system (CNS) by infected cells [52]. Elevated Tat mRNA levels have been detected in the brain of AIDS patients [53], where Tat is believed to play a significant role in the pathogenesis of HAD through not only its direct neurotoxicity, but also through the release of deleterious products in microglial cells [54]. Although they act as CNS macrophages, microglia cells differ in many aspects from peripheral macrophages. Their morphological and functional specificity responds to cell-cell contacts and secreted factors from surrounding astrocytes and neurons. The strict separation of microglia cells from blood components is due to the blood brain barrier (BBB). This results in a down-regulated "surveillance" phenotype [55]. Microglial cells are nevertheless able to undergo activation and acquire typical macrophage functions such as phagocytosis of microbes or apoptotic bodies and the secretion of inflammatory or anti-inflammatory mediators [56,57].

Tat activates microglia and impairs major molecular mechanisms that normally prevent or shorten microglial activation. As is the case in macrophages, Tat increases microglial production of free radicals as well as pro-inflammatory cytokines and chemokines [42,43,58,59]. Tat induction of NO and inducible NO synthase (iNOS) is enhanced by IFN-γ [60]. This suggests that Tat and IFN-γ cooperatively contribute to the severity of brain damage observed in brain tissues from AIDS patients and animal HAD models.

The transcription factor NF-κB plays a central role in the regulation of inflammatory gene expression and is involved in most Tat-induced effects in microglial cultures [61]. In surveillance microglia, signals provided by astrocytes actively contribute to NF-κB down-modulation [62]. Elevated immunoreactivity for p50/p65 heterodimer subunits was found in microglia and brain macrophages of children with HIV encephalitis [63] despite repression by the surrounding cells. Likewise, nuclear staining for NF-κB in the perivascular microglia/macrophages of deep white matter and basal ganglia correlated with the severity of HIV-associated dementia in AIDS patients [64]. Interestingly, Tat-induced formation of free radicals in microglial cells occurs independently from NF-κB activation [65,66], as lipid peroxidation and oxidative stress still occur in microglial cultures exposed to Tat in the presence of NF-κB inhibitors [65]. Likewise, the pro-oxidant activities of Tat in the N9 microglial cell line depend on MAP kinase activation [66]. Additionally, antioxidants abrogate oxidative stress rather than the other Tat-induced functions such as IL-1β, NO, and TNF-α production or IkBα degradation [65]. Thus, Tat-induced NF-κB activation in microglia may not require the formation of free radicals, although oxidative stress is contributive to its activation [67].

In different cell types, including macrophages and microglia, Tat influences cell function by modifying Ca^2+ ^homeostasis [43]. Indeed, Tat possesses a cysteine-cysteine-phenylalanine domain, enabling Tat to mimic beta chemokine effects on both Ca^2+ ^movements and chemotaxis [38]. In microglia, Ca^2+ ^mobilization and cell migration by Tat are sensitive to pertussis toxin (PTX), but not cholera toxin. This observation supports the involvement of Gi rather than Gs type proteins, as expected for chemokine receptor stimulation [37]. Furthermore, cross-desensitization studies revealed CCR3 receptor involvement. Similar to findings in monocytes, Tat-induced Ca^2+ ^signals in human microglia are characterized by rapid desensitization [68].

Nanomolar concentrations of recombinant Tat have been shown to decrease in a dose- and time-dependent manner, cAMP accumulation induced in microglial cultures by the β-adrenergic receptor agonist isoproterenol, or by forskolin, an activator of adenylyl cyclase [69]. In microglia, increased cAMP accumulation lowers potentially neurotoxic pro-inflammatory molecules [70-76] and promotes the production of neuroprotective or immunosuppressive substances [70]. Thus, Tat may interfere with cAMP's control on microglial activation.

Among the ion channels expressed by microglial cells, there are two major classes of K+-permeable channels: the delayed-outward-rectifying (Kdr) and the inward-rectifying (Kir) channels. Their expression differs in macrophages and microglia. Their expression is finely modulated by both activation and differentiation [77-81]. Chronic microglial cell treatment with high Tat concentration (≥ 100 ng/mL) up-regulates Kdr currents due to NF-κB-dependent increase in channel expression without a significant increase in Kdr currents [82]. Therefore, the hyperpolarization thus induced by Tat may has several consequences. Ca^2+ ^influx depends on a hyperpolarized membrane potential and Tat's β-chemokine mimicry may thus be favored by Kdr currents. Kdr currents may also modulate the microglial respiratory burst and the transport of amino acids through voltage-dependent transporters. The latter is likely to modify the availability of amino acids for protein synthesis [83,84], as well as the dynamics of glutamate exchange between intracellular and extracellular pools. This may affect the regulation of both extracellular glutamate concentration in the vicinity of glutamate-sensitive neurons and glutathione synthesis rate in microglia [85-87].

### Vpr

Vpr is a 96 amino acid-long virion-associated protein located in the cytoplasm and nucleus of HIV-infected cells [88-93]. Vpr is not essential for viral replication in T cells, but critical for HIV replication in non-dividing cells such as macrophages [94-99]. Vpr has pleiotropic effects on viral replication, cellular proliferation and differentiation, cytokine production, NF-κB-mediated transcription and apoptosis [100-103].

Vpr has been shown to induce cell cycle arrest at the G2 cell cycle phase [104-107]. G2 cell cycle arrest correlates with the inhibition of Cdc2 activity and parallels enhanced viral replication [108-110]. G2 cell cycle arrest is followed by apoptosis in HIV-infected and Vpr-expressing cells [111]. Apoptosis is mediated through the interaction of Vpr with the mitochondrion permeability transition pore. This interaction opens the pore, causing mitochondrial swelling, release of cytochrome C as well as caspase 9 and caspase 3 activation [111]. p53 tumor suppressor protein may be implicated in cell cycle arrest and apoptosis mediated by Vpr in certain cell types [107].

Vpr transactivates the viral promoter and HIV-1 LTR resulting in increased viral replication. The G2 cell cycle arrest is concomitant with high levels of viral replication in primary human CD4+ T cells. An interaction between Vpr, Sp1 and TFIIB transcription factors is required for Vpr-mediated transcriptional enhancement of HIV-1 LTR [112,113].

Vpr-mediated transactivation necessitates intact NF-κB sites and depends on Vpr's ability to stimulate p300/CBP coactivator function, which promotes cooperative interaction between the RelA subunit of NF-κB and the cyclin B1Cdc2 [114]. A structural and functional interaction between Vpr and Tat has been reported, synergistically enhancing the transcriptional activity of the HIV-1 LTR [114].

The activity of recombinant Vpr (rVpr) in macrophages has been investigated. High concentrations of rVpr as well as the carboxy-terminal Vpr peptide are cytotoxic to macrophages. However, at low concentrations rVpr was shown to enhance the activity of several transcription factors including AP-1, c-Jun, and, NF-κB [115]. Amino- and carboxy-terminal Vpr peptides retained transcription factor activation properties, albeit to a lesser extent than with the full-length rVpr. Similarly to Vpr expressed in infected cells, rVpr stimulated HIV-1 replication in acutely infected primary macrophages. Furthermore, reduced p24 production by macrophages infected with Vpr-deficient virus could be rescued by adding rVpr to culture medium [116]. Exposure to rVpr also increased transcription and p21/waf1 levels in macrophages [117]. These Vpr effects on macrophages may reflect the mechanisms by which Vpr activates the HIV-1 LTR and enhances virus replication in acutely and latently infected cells [88]. Although primarily considered to be a regulator of viral promoter transactivation, transcription factor activation may have significant effects on macrophage cellular functions [117,118]. Additionally, macrophages and PBLs produce less chemokines following recombinant Vpr treatment. This observation suggests that Vpr modulates cytokine production by interfering with NF-κB-mediated transcription [119,120].

### gp120

HIV-1 infects human T cells and monocytes/macrophages through the interaction of gp120 with CD4 and the CXCR4 or CCR5 co-receptor, which determines the cellular tropism [121-131]. HIV-1 gp120 down-regulates CD4 expression in primary human macrophages through induction of endogenous TNFα [121,132-136]. Actually, TNFα down-regulates both surface and total CD4 expression in primary human macrophages at the transcription level [134,137-140]. TNFα inhibits R5 and R5/X4 HIV-1 entry into primary macrophages via downregulation of both cell surface CD4 and CCR5 and via enhanced secretion of CC-chemokines, MIP-1α, MIP-1β and RANTES [129,137,141-146]. An iterative pretreatment of primary macrophages with TNFα prior to HIV infection inhibits HIV-1 replication in primary macrophages [142]. The inhibition of HIV-1 entry into primary macrophages following TNFα pretreatment involves TNFR2 and is mediated by the secretion of CC-chemokines such as RANTES, MIP-1α, and MIP-1β[140,141]. TNFα induces the production of RANTES, MIP-1α, and MIP-1β, which in turn down-regulate cell surface CCR5 expression on primary macrophages, resulting in the inhibition of R5 HIV-1 entry [147-151]. In agreement with this observation RANTES inhibits HIV-1 envelope-mediated membrane fusion in primary macrophages [152] and inhibits the activity of the RANTES promoter containing four NF-κB binding sites which is up-regulated by TNFα [153].

Many studies conducted over the past two decades have shown that besides infection, exposure of macrophages to intact virions or soluble gp120 may exert various functional effects on macrophages, including cytokine secretion activation [121,135,154]. However, the specific pathways involved in gp120-induced responses have only been defined recently. The presence of non-infectious virion particles in excess of infectious virus, the ability of gp120 to dissociate from the transmembrane gp41 portion of Env as well as detection of circulating gp120 in infected patients [155] have raised the question of what biological activities this protein is involved in aside from mediating infection. Such studies have demonstrated the ability of gp120 to activate intracellular signaling in multiple cell types as a result of its binding to receptor/co-receptor complex. Although gp120-induced signaling has been extensively investigated in CD4+ T cells, gp120 has also been reported to activate intracellular signals in macrophages [156].

In primary human macrophages, both R5 and X4 gp120 induce calcium mobilization, although R5 gp120 elicited higher peaks and more sustained elevations than X4 gp120 [157,158]. Single-cell patch-clamp recording combined with pharmacological antagonists and current reversal potential analysis identified the ion channels associated with CCR5 and CXCR4 activation: chloride, calcium-activated potassium, and non-selective cation (NSC) channels [157]. These responses to HIV-1 gp120 were mediated by chemokine receptors, but not by CD4, since the responses to R5 Env were absent in macrophages from patients lacking cell surface CCR5 expression (CCR5Δ 32); responses to X4 gp120 were inhibited by a small molecule CXCR4 antagonist [157,159]. While R5 and X4 gp120 generally induced similar signals through CCR5 and CXCR4, respectively, certain differences were noted. R5 Env opened the calcium-activated outward K^+ ^channels more frequently than X4 gp120, and induced Cl- currents of greater amplitude. Gp120, instead of CXCR4 or CCR5 binding chemokines, activated the NSC channel [160].

In addition, gp120 has been shown to activate all three MAPK family members (ERK1/2, JNK, and p38) in macrophages. R5 gp120 triggered macrophage release of MIP-1, MCP-1, and TNFα. The secretion of these products was blocked by small molecule inhibitors of ERK1/2 and p38 MAPKs [39,161].

The src kinases Lyn and Hck are highly expressed in macrophages, and recent *in vitro *kinase assays demonstrated that R5 gp120 and MIP-1β activated Lyn in macrophages [162]. Neither R5 gp120 nor MIP-1β activated Lyn in macrophages derived from CCR5Δ 32 donors or in cells treated with a small molecule CCR5 inhibitor, indicating that Lyn activation was elicited through CCR5 receptor. Unlike Lyn, Hck activation did not occur in response to gp120 or chemokine stimulation [162,163]. Both a Lyn-specific peptide pseudo-substrate inhibitor and PP2, a broad src family kinase inhibitor, suppressed gp120-induced TNFα production. These results are suggestive of a signaling cascade initiated by gp120 through CCR5, involving Lyn activation of the MAPK pathway, resulting in gp120-induced TNFα release.

Several lines of evidence indicate that HIV-1 gp120/chemokine receptor interactions activate PI3K in macrophages [39,164]. This finding is based upon R5 gp120 activation of protein kinase B (PKB), a downstream target for class I PI3K and a useful indirect indicator of its activation. Furthermore, several small molecule PI3K inhibitors blocked gp120-induced CCR5-mediated ERK1/2 and p38 phosphorylation, as well as TNFα release. These results not only suggest a role for PI3Ks in CCR5 signaling but also indicate that, like Lyn, PI3K acts upstream of MAPKs in the regulation of cytokine production through this pathway [39]. It is unclear which PI3K isoform is involved in these R5 gp120-induced signals, and the relationship between PI3K and Lyn remains to be determined.

Besides chemokine receptors, interactions between HIV-1 gp120 and CD4 stimulate signal transduction pathways, such as activation of PKC, generation of PKC-dependent phosphorylation of CD4, and activation of the ERK/MAPK pathway, which in turn stimulates transcription factors such as NF-kB, AP-1, and Elk-1, as well as induction of cytokine and chemokine gene expression [115,165-172]. Early inflammatory gene products such as TNFα, may stimulate HIV-1 replication in the absence of HIV-1 Tat protein. Thus, the activation of cellular signaling pathways leading to the production of cytokine and chemokine genes by HIV-1 gp120 could facilitate viral replication in the early phases of the viral life cycle [50].

Proline-rich tyrosine kinase 2 (Pyk2) activation has been suggested as a critical signalling mechanism for integrin-mediated formation of adhesion contacts in macrophages known as podosomes. Pyk2 is known to be activated by chemokines, triggering cell migration [173,174]. CCR5 and CXCR4 are both linked to Pyk2, which is activated by R5 gp120 and MIP-1β as well as X4 gp120 and SDF-1α [161]. Recently, a functional role for Pyk2 in the migration of macrophages has been demonstrated using Pyk2 knockout mice [175], suggesting that gp120 may be involved in macrophage migration.

## Macrophage Signaling and HIV-1 Pathogenesis

In this section, we report that several HIV-1 proteins may modulate the macrophage signaling pathway resulting in T lymphocytes depletion and viral cellular reservoir formation, especially in macrophages [176].

### Macrophage signaling and T cell apoptosis

Increased spontaneous and activation-induced apoptosis of peripheral CD4+ T cells from HIV-infected patients is observed *ex vivo *in lymph nodes of HIV-infected patients and of SIV-infected macaques [177-180]. Deciphering the molecular mechanisms involved in CD4+ T cell apoptosis in HIV-infected patients is critical to understanding HIV pathogenesis.

**Figure 2:**
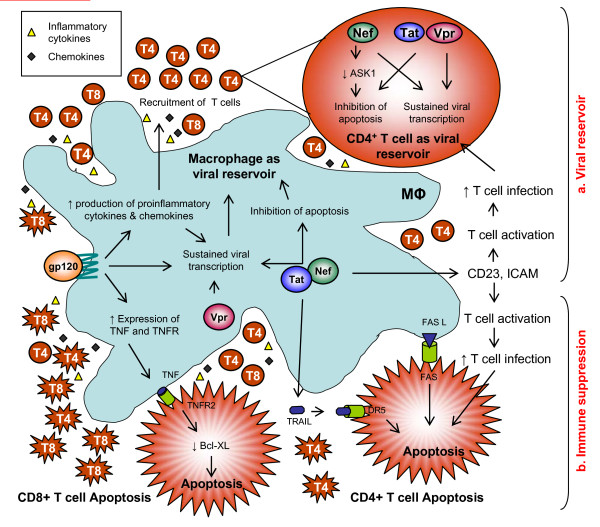
**A model of HIV-1 pathogenesis based on interactions between macrophages and T cells which account for increased immune suppression and cellular virion reservoirs**. a) Viral glycoprotein gp120 activates the production of pro-inflammatory cytokines and chemokines by macrophages, attracting T cells in the vicinity of macrophages, thereby increasing the number of infected cells and fueling the viral reservoirs. HIV-1 proteins Nef, Tat, and Vpr activate the long terminal repeat (LTR) of HIV-1, resulting in sustained viral growth while also activating anti-apoptotic pathways that favor viral persistence and formation of viral reservoir. b) Viral protein Tat participates in CD4+ T cell death through TRAIL secretion by HIV-1 infected macrophages. Viral gp120 glycoproteins increase the expression of TNF and TNFR on macrophages and T cells, leading to CD8+ T cell apoptosis. Thus, macrophage signaling using viral proteins accounts for both viral persistence and immune suppression during HIV-1 infection.

In macrophages, Nef has been shown to activate multiple cellular pathways, possibly leading to increased infection of adjacent T cells through bystander mechanisms involving T cell activation (Figure 2). It has been shown that Nef-expressing macrophages enhance resting CD4+ T cell permissiveness through a complex cellular and soluble interaction involving macrophages, B cells, and CD4+ T cells [29]. Nef expression within macrophages via adenoviral vectors has been shown to induce the secretion of soluble CD23 and ICAM, resulting in up-regulation of costimulatory B cell receptors, including CD22, CD54, CD58, and CD80. This leads to T cell activation upon interaction with B cells via these costimulatory receptors, thus enabling the generation of non-productive or productive reservoirs, depending on the interactions [29].

Furthermore, Nef has been reported to prevent Fas- and TNF-receptor-mediated deaths observed in HIV-infected T cells via interaction with the apoptosis signal regulating kinase-1 (ASK-1). Nef inhibits ASK-1, caspase 3 and caspase 8 activation, resulting in apoptosis blockade in HIV-infected cells [181-184]. Apoptosis was measured in productively infected CD4+ T lymphocytes using a reporter virus and a recombinant HIV infectious clone expressing the green fluorescent protein (GFP) in the presence and absence of autologous macrophages. The survival of productively infected CD4+ T lymphocytes has been shown to require Nef expression and activation by TNFα expressed on macrophage surface, thereby participating in the formation and maintenance of viral reservoirs in HIV-infected patients [184].

In addition to the macrophage-mediated formation of T cell reservoirs, *in vitro *culture models demonstrate that uninfected CD4+ T cells undergo apoptosis upon contact with HIV-infected cells; for example mononuclear phagocytes [180]. Macrophages play a major role in this process, suggesting that apoptosis-inducing ligands expressed by macrophages mediate apoptosis of susceptible CD4+ T cells [159,185-187]. Activated macrophages produce TNFα following HIV infection *in vitro *[135]. TNFα is released as a soluble factor or expressed on the surface of macrophages under a membrane-bound form that primarily targets TNFR2 rather than TNFR1 [188,189]. TNFR2 stimulation may trigger T cell apoptosis, especially in CD8+ T cells [188]. TNFα and TNF receptors are increased in HIV-infected patients and inversely correlated with CD4+ T cell counts [190]. TNFα is expressed on the surface of activated macrophages, and cell surface TNFR2 is not increased on CD4+ infected T cells. Therefore, for the most part, the apoptosis of CD4+ T lymphocytes is mediated via Fas/Fas ligand interaction [185,186,191]. TNFα causes death at a later stage than Fas and may be transduced through TNFR2, which does not contain homology to the Fas death domain and uses different signaling pathways than TNFR1 [115,185]. Recently, Tat has been reported to induce secretion of soluble TNF-related apoptosis-induced ligand (TRAIL) in human macrophages, leading to the death of bystander CD4+ T lymphocytes [73]. Thus, the production of TRAIL by Tat-stimulated monocytes/macrophages is likely to be an additional mechanism by which HIV-1 infection destroys uninfected bystander cells.

CD8+ T cell apoptosis during HIV infection has been shown to result from the interaction between membrane-bound TNFα expressed on the surface of activated macrophages and TNFR2 expressed on the surface of activated CD8+ T cells [158]. Both membrane-bound TNFα and TNFR2 are up-regulated on macrophages and CD8+ T cells, respectively, following CXCR4 stimulation by HIV gp120. However, CCR5 may also play a role, albeit minor [158]. TNFR2 stimulation of T cells results in decreased intracellular levels of apoptosis protective protein Bcl-XL, a member of the Bcl-2 family [192]. Impaired induction of Bcl-XL has been observed in PBMC isolated from HIV-infected patients [193]. Therefore, TNFR2 stimulation of CD8+ T cells by membrane-bound TNFα expressed on the surface of macrophages might decrease the intracellular levels of anti-apoptotic proteins resulting in CD8+ T cell death.

Additionally, chemokines and activated macrophages have been reported to play a role in HIV-1 gp120-induced neuronal apoptosis [194,195].

### Macrophage signaling and formation of viral reservoirs

Whereas CD4+ T cells die within a few days after becoming infected with HIV, infected macrophages seem to persist for months, continuing to release viruses. Several reasons may explain why macrophages are a major cellular reservoir of virions during infection (Figure 2). Macrophages are more resistant than T cells to HIV-induced apoptosis and therefore allow for sustained viral production without fatal cell death. Persistent HIV infection of macrophages results in increased NF-κB levels, involved in the resistance to TNFα-induced apoptosis. Macrophages release CC-chemokines which have the ability to attract CD4+ and CD8+ T lymphocytes in their vicinity [196]. They may also block the entry of R5 HIV-1 virions into CD4+ target cells [122]. CC-chemokine production is often associated with that of pro-inflammatory cytokines, such as TNFα and IL-1β, which stimulate the transcription of HIV LTR via activation of NF-kB [197,198]. Additionally, TNFα may block entry of R5 HIV-1 strains into macrophages via a decreased expression of CCR5 on cell surfaces [137,141,142,147]. Thus, CC-chemokines and pro-inflammatory cytokines facilitate the recruitment and productive infection of CD4+ T lymphocytes via increased viral transcription, while regulating the entry of virions into macrophages, thereby preventing macrophage superinfection. Additionally, apoptosis inhibition in HIV-1 infected T cells enhances virus production and facilitates persistent infection [199]. HIV-1 proteins, by modulation of the TNFR signaling pathway, lead to the formation of viral reservoirs, especially in primary macrophages [50]. Altogether, the data indicate that both viral and cellular factors are involved in the controlled and sustained production of virions in infected CD4+ T lymphocytes and macrophages, thereby expanding the viral reservoir which fuels disease progression.

## Conclusion

The macrophage is essential in the loss of T lymphocytes and formation of viral reservoirs; it plays a critical role in HIV-1 disease progression. Several HIV-1 proteins modulate signaling in infected and bystander macrophages, thereby facilitating disease progression. A better understanding of the manner by which HIV-1 modulates signaling in macrophages may be instrumental in the development of new therapeutic approaches that may ultimately restrict or decrease the size of cellular virion reservoirs in HIV-1-infected patients.

## Competing interests

The authors declare that they have no competing interests.

## Authors' contributions

GH was responsible for drafting and revising the manuscript as well as organizing the content. GG was responsible for drafting and revising the section "Action of Tat on microglia". KAK created Figures 1 and 2. WA assisted in revising the manuscript.

## References

[B1] Coleman CM Wu L HIV interactions with monocytes and dendritic cells: viral latency and reservoirs Retrovirology 2009 6 51 10.1186/1742-4690-6-51 19486514 PMC2697150

[B2] Foster JL Garcia JV HIV-1 Nef: at the crossroads Retrovirology 2008 5 84 10.1186/1742-4690-5-84 18808677 PMC2563024

[B3] Aiken C Trono D Nef stimulates human immunodeficiency virus type 1 proviral DNA synthesis J Virol 1995 69 5048 5056 7541845 10.1128/jvi.69.8.5048-5056.1995 PMC189322

[B4] Bazan JF Bacon KB Hardiman G Wang W Soo K Rossi D Greaves DR Zlotnik A Schall TJ A new class of membrane-bound chemokine with a CX3C motif Nature 1997 385 640 644 10.1038/385640a0 9024663

[B5] Stein M Keshav S Harris N Gordon S Interleukin 4 potently enhances murine macrophage mannose receptor activity: a marker of alternative immunologic macrophage activation J Exp Med 1992 176 287 292 10.1084/jem.176.1.287 1613462 PMC2119288

[B6] Miller MD Warmerdam MT Gaston I Greene WC Feinberg MB The human immunodeficiency virus-1 nef gene product: a positive factor for viral infection and replication in primary lymphocytes and macrophages J Exp Med 1994 179 101 113 10.1084/jem.179.1.101 8270859 PMC2191317

[B7] Lama J Mangasarian A Trono D Cell-surface expression of CD4 reduces HIV-1 infectivity by blocking Env incorporation in a Nef- and Vpu-inhibitable manner Curr Biol 1999 9 622 631 10.1016/S0960-9822(99)80284-X 10375528

[B8] Schwartz O Marechal V Le Gall S Lemonnier F Heard JM Endocytosis of major histocompatibility complex class I molecules is induced by the HIV-1 Nef protein Nat Med 1996 2 338 342 10.1038/nm0396-338 8612235

[B9] Fujii Y Otake K Tashiro M Adachi A Soluble Nef antigen of HIV-1 is cytotoxic for human CD4+ T cells FEBS Lett 1996 393 93 96 10.1016/0014-5793(96)00859-9 8804432

[B10] Brigino E Haraguchi S Koutsonikolis A Cianciolo GJ Owens U Good RA Day NK Interleukin 10 is induced by recombinant HIV-1 Nef protein involving the calcium/calmodulin-dependent phosphodiesterase signal transduction pathway Proc Natl Acad Sci USA 1997 94 3178 3182 10.1073/pnas.94.7.3178 9096366 PMC20342

[B11] Qiao X He B Chiu A Knowles DM Chadburn A Cerutti A Human immunodeficiency virus 1 Nef suppresses CD40-dependent immunoglobulin class switching in bystander B cells Nat Immunol 2006 7 302 310 10.1038/ni1302 16429138

[B12] Tobiume M Fujinaga K Suzuki S Komoto S Mukai T Ikuta K Extracellular Nef protein activates signal transduction pathway from Ras to mitogen-activated protein kinase cascades that leads to activation of human immunodeficiency virus from latency AIDS Res Hum Retroviruses 2002 18 461 467 10.1089/088922202753614227 11958689

[B13] Varin A Manna SK Quivy V Decrion AZ Van Lint C Herbein G Aggarwal BB Exogenous Nef protein activates NF-kappa B, AP-1, and c-Jun N-terminal kinase and stimulates HIV transcription in promonocytic cells. Role in AIDS pathogenesis J Biol Chem 2003 278 2219 2227 10.1074/jbc.M209622200 12419805

[B14] Alessandrini L Santarcangelo AC Olivetta E Ferrantelli F d'Aloja P Pugliese K Pelosi E Chelucci C Mattia G Peschle C Verani P Federico M T-tropic human immunodeficiency virus (HIV) type 1 Nef protein enters human monocyte-macrophages and induces resistance to HIV replication: a possible mechanism of HIV T-tropic emergence in AIDS J Gen Virol 2000 81 2905 2917 11086122 10.1099/0022-1317-81-12-2905

[B15] Quaranta MG Camponeschi B Straface E Malorni W Viora M Induction of interleukin-15 production by HIV-1 nef protein: a role in the proliferation of uninfected cells Exp Cell Res 1999 250 112 121 10.1006/excr.1999.4494 10388525

[B16] Quaranta MG Mattioli B Spadaro F Straface E Giordani L Ramoni C Malorni W Viora M HIV-1 Nef triggers Vav-mediated signaling pathway leading to functional and morphological differentiation of dendritic cells FASEB J 2003 17 2025 2036 10.1096/fj.03-0272com 14597672

[B17] Kuster H Opravil M Ott P Schlaepfer E Fischer M Gunthard HF Luthy R Weber R Cone RW Treatment-induced decline of human immunodeficiency virus-1 p24 and HIV-1 RNA in lymphoid tissue of patients with early human immunodeficiency virus-1 infection Am J Pathol 2000 156 1973 1986 10854220 10.1016/S0002-9440(10)65070-5 PMC1850084

[B18] Soudeyns H Rebai N Pantaleo GP Ciurli C Boghossian T Sekaly RP Fauci AS The T cell receptor V beta repertoire in HIV-1 infection and disease Semin Immunol 1993 5 175 185 10.1006/smim.1993.1021 8102262

[B19] Olivetta E Percario Z Fiorucci G Mattia G Schiavoni I Dennis C Jager J Harris M Romeo G Affabris E Federico M HIV-1 Nef induces the release of inflammatory factors from human monocyte/macrophages: involvement of Nef endocytotic signals and NF-kappa B activation J Immunol 2003 170 1716 1727 12574335 10.4049/jimmunol.170.4.1716

[B20] Federico M Percario Z Olivetta E Fiorucci G Muratori C Micheli A Romeo G Affabris E HIV-1 Nef activates STAT1 in human monocytes/macrophages through the release of soluble factors Blood 2001 98 2752 2761 10.1182/blood.V98.9.2752 11675348

[B21] Percario Z Olivetta E Fiorucci G Mangino G Peretti S Romeo G Affabris E Federico M Human immunodeficiency virus type 1 (HIV-1) Nef activates STAT3 in primary human monocyte/macrophages through the release of soluble factors: involvement of Nef domains interacting with the cell endocytotic machinery J Leukoc Biol 2003 74 821 832 10.1189/jlb.0403161 12960275

[B22] Mangino G Percario ZA Fiorucci G Vaccari G Manrique S Romeo G Federico M Geyer M Affabris E In vitro treatment of human monocytes/macrophages with myristoylated recombinant Nef of human immunodeficiency virus type 1 leads to the activation of mitogen-activated protein kinases, IkappaB kinases, and interferon regulatory factor 3 and to the release of beta interferon J Virol 2007 81 2777 2791 10.1128/JVI.01640-06 17182689 PMC1865981

[B23] Schrager JA Der Minassian V Marsh JW HIV Nef increases T cell ERK MAP kinase activity J Biol Chem 2002 277 6137 6142 10.1074/jbc.M107322200 11726657

[B24] Hiscott J Pitha P Genin P Nguyen H Heylbroeck C Mamane Y Algarte M Lin R Triggering the interferon response: the role of IRF-3 transcription factor J Interferon Cytokine Res 1999 19 1 13 10.1089/107999099314360 10048763

[B25] Sato M Tanaka N Hata N Oda E Taniguchi T Involvement of the IRF family transcription factor IRF-3 in virus-induced activation of the IFN-beta gene FEBS Lett 1998 425 112 116 10.1016/S0014-5793(98)00210-5 9541017

[B26] Yoneyama M Suhara W Fukuhara Y Fukuda M Nishida E Fujita T Direct triggering of the type I interferon system by virus infection: activation of a transcription factor complex containing IRF-3 and CBP/p300 EMBO J 1998 17 1087 1095 10.1093/emboj/17.4.1087 9463386 PMC1170457

[B27] Kilareski EM Shah S Nonnemacher MR Wigdahl B Regulation of HIV-1 transcription in cells of the monocyte-macrophage lineage Retrovirology 2009 6 118 10.1186/1742-4690-6-118 20030845 PMC2805609

[B28] Herbein G Varin A Larbi A Fortin C Mahlknecht U Fulop T Aggarwal BB Nef and TNFalpha are coplayers that favor HIV-1 replication in monocytic cells and primary macrophages Curr HIV Res 2008 6 117 129 10.2174/157016208783884985 18336259

[B29] Swingler S Brichacek B Jacque JM Ulich C Zhou J Stevenson M HIV-1 Nef intersects the macrophage CD40L signalling pathway to promote resting-cell infection Nature 2003 424 213 219 10.1038/nature01749 12853962 PMC9524218

[B30] Mahlknecht U Will J Varin A Hoelzer D Herbein G Histone deacetylase 3, a class I histone deacetylase, suppresses MAPK11-mediated activating transcription factor-2 activation and represses TNF gene expression J Immunol 2004 173 3979 3990 15356147 10.4049/jimmunol.173.6.3979

[B31] Jones KA Tat and the HIV-1 promoter Curr Opin Cell Biol 1993 5 461 468 10.1016/0955-0674(93)90012-F 8352964

[B32] Jeang KT Xiao H Rich EA Multifaceted activities of the HIV-1 transactivator of transcription, Tat J Biol Chem 1999 274 28837 28840 10.1074/jbc.274.41.28837 10506122

[B33] Ensoli B Buonaguro L Barillari G Fiorelli V Gendelman R Morgan RA Wingfield P Gallo RC Release, uptake, and effects of extracellular human immunodeficiency virus type 1 Tat protein on cell growth and viral transactivation J Virol 1993 67 277 287 8416373 10.1128/jvi.67.1.277-287.1993 PMC237361

[B34] Noonan D Albini A From the outside in: extracellular activities of HIV Tat Adv Pharmacol 2000 48 229 250 full_text 10987093 10.1016/s1054-3589(00)48008-7

[B35] Gautier VW Gu L O'Donoghue N Pennington S Sheehy N Hall WW In vitro nuclear interactome of the HIV-1 Tat protein Retrovirology 2009 6 47 10.1186/1742-4690-6-47 19454010 PMC2702331

[B36] Chang HK Gallo RC Ensoli B Regulation of Cellular Gene Expression and Function by the Human Immunodeficiency Virus Type 1 Tat Protein J Biomed Sci 1995 2 189 202 10.1007/BF02253380 11725056

[B37] Albini A Benelli R Giunciuglio D Cai T Mariani G Ferrini S Noonan DM Identification of a novel domain of HIV tat involved in monocyte chemotaxis J Biol Chem 1998 273 15895 15900 10.1074/jbc.273.26.15895 9632634

[B38] Xiao H Neuveut C Tiffany HL Benkirane M Rich EA Murphy PM Jeang KT Selective CXCR4 antagonism by Tat: implications for in vivo expansion of coreceptor use by HIV-1 Proc Natl Acad Sci USA 2000 97 11466 11471 10.1073/pnas.97.21.11466 11027346 PMC17223

[B39] Cardona AE Pioro EP Sasse ME Kostenko V Cardona SM Dijkstra IM Huang D Kidd G Dombrowski S Dutta R Lee JC Cook DN Jung S Lira SA Littman DR Ransohoff RM Control of microglial neurotoxicity by the fractalkine receptor Nat Neurosci 2006 9 917 924 10.1038/nn1715 16732273

[B40] Lafrenie RM Wahl LM Epstein JS Hewlett IK Yamada KM Dhawan S HIV-1-Tat modulates the function of monocytes and alters their interactions with microvessel endothelial cells. A mechanism of HIV pathogenesis J Immunol 1996 156 1638 1645 8568270

[B41] Lafrenie RM Wahl LM Epstein JS Hewlett IK Yamada KM Dhawan S HIV-1-Tat protein promotes chemotaxis and invasive behavior by monocytes J Immunol 1996 157 974 977 8757599

[B42] Nimmerjahn A Kirchhoff F Helmchen F Resting microglial cells are highly dynamic surveillants of brain parenchyma in vivo Science 2005 308 1314 1318 10.1126/science.1110647 15831717

[B43] Power C McArthur JC Nath A Wehrly K Mayne M Nishio J Langelier T Johnson RT Chesebro B Neuronal death induced by brain-derived human immunodeficiency virus type 1 envelope genes differs between demented and nondemented AIDS patients J Virol 1998 72 9045 9053 9765449 10.1128/jvi.72.11.9045-9053.1998 PMC110321

[B44] Puri RK Aggarwal BB Human immunodeficiency virus type 1 tat gene up-regulates interleukin 4 receptors on a human B-lymphoblastoid cell line Cancer Res 1992 52 3787 3790 1617647

[B45] Rautonen J Rautonen N Martin NL Wara DW HIV type 1 Tat protein induces immunoglobulin and interleukin 6 synthesis by uninfected peripheral blood mononuclear cells AIDS Res Hum Retroviruses 1994 10 781 785 7986583 10.1089/aid.1994.10.781

[B46] Sastry KJ Reddy HR Pandita R Totpal K Aggarwal BB HIV-1 tat gene induces tumor necrosis factor-beta (lymphotoxin) in a human B-lymphoblastoid cell line J Biol Chem 1990 265 20091 20093 2243081

[B47] Puri RK Leland P Aggarwal BB Constitutive expression of human immunodeficiency virus type 1 tat gene inhibits interleukin 2 and interleukin 2 receptor expression in a human CD4+ T lymphoid (H9) cell line AIDS Res Hum Retroviruses 1995 11 31 40 10.1089/aid.1995.11.31 7734194

[B48] Husain SR Leland P Aggarwal BB Puri RK Transcriptional up-regulation of interleukin 4 receptors by human immunodeficiency virus type 1 tat gene AIDS Res Hum Retroviruses 1996 12 1349 1359 10.1089/aid.1996.12.1349 8891114

[B49] Lotz M Clark-Lewis I Ganu V HIV-1 transactivator protein Tat induces proliferation and TGF beta expression in human articular chondrocytes J Cell Biol 1994 124 365 371 10.1083/jcb.124.3.365 8294518 PMC2119928

[B50] Herbein G Khan KA Is HIV infection a TNF receptor signalling-driven disease? Trends Immunol 2008 29 61 67 10.1016/j.it.2007.10.008 18178131

[B51] Kumar A Manna SK Dhawan S Aggarwal BB HIV-Tat protein activates c-Jun N-terminal kinase and activator protein-1 J Immunol 1998 161 776 781 9670954

[B52] Ensoli B Buonaguro L Barillari G Fiorelli V Gendelman R Morgan RA Wingfield P Gallo RC Release, uptake, and effects of extracellular human immunodeficiency virus type 1 Tat protein on cell growth and viral transactivation J Virol 1993 67 277 287 8416373 10.1128/jvi.67.1.277-287.1993 PMC237361

[B53] Nath A Geiger J Neurobiological aspects of human immunodeficiency virus infection: neurotoxic mechanisms Prog Neurobiol 1998 54 19 33 10.1016/S0301-0082(97)00053-1 9460791

[B54] Ghafouri M Amini S Khalili K Sawaya BE HIV-1 associated dementia: symptoms and causes Retrovirology 2006 3 28 10.1186/1742-4690-3-28 16712719 PMC1513597

[B55] Ransohoff RM Perry VH Microglial physiology: unique stimuli, specialized responses Annu Rev Immunol 2009 27 119 145 10.1146/annurev.immunol.021908.132528 19302036

[B56] Minghetti L Levi G Microglia as effector cells in brain damage and repair: focus on prostanoids and nitric oxide Prog Neurobiol 1998 54 99 125 10.1016/S0301-0082(97)00052-X 9460796

[B57] Streit WJ Microglia and the response to brain injury Ernst Schering Res Found Workshop 2002 11 24 12066409 10.1007/978-3-662-05073-6_2

[B58] Sheng WS Hu S Hegg CC Thayer SA Peterson PK Activation of human microglial cells by HIV-1 gp41 and Tat proteins Clin Immunol 2000 96 243 251 10.1006/clim.2000.4905 10964543

[B59] Sawaya BE Thatikunta P Denisova L Brady J Khalili K Amini S Regulation of TNFalpha and TGFbeta-1 gene transcription by HIV-1 Tat in CNS cells J Neuroimmunol 1998 87 33 42 10.1016/S0165-5728(98)00044-7 9670843

[B60] Polazzi E Levi G Minghetti L Human immunodeficiency virus type 1 Tat protein stimulates inducible nitric oxide synthase expression and nitric oxide production in microglial cultures J Neuropathol Exp Neurol 1999 58 825 831 10.1097/00005072-199908000-00005 10446807

[B61] De Simone R Ajmone-Cat MA Minghetti L Atypical antiinflammatory activation of microglia induced by apoptotic neurons: possible role of phosphatidylserine-phosphatidylserine receptor interaction Mol Neurobiol 2004 29 197 212 10.1385/MN:29:2:197 15126686

[B62] Rosenstiel P Lucius R Deuschl G Sievers J Wilms H From theory to therapy: implications from an in vitro model of ramified microglia Microsc Res Tech 2001 54 18 25 10.1002/jemt.1116 11526952

[B63] Dollard SC James HJ Sharer LR Epstein LG Gelbard HA Dewhurst S Activation of nuclear factor kappa B in brains from children with HIV-1 encephalitis Neuropathol Appl Neurobiol 1995 21 518 528 10.1111/j.1365-2990.1995.tb01098.x 8745241

[B64] Rostasy K Monti L Yiannoutsos C Wu J Bell J Hedreen J Navia BA NFkappaB activation, TNF-alpha expression, and apoptosis in the AIDS-Dementia-Complex J Neurovirol 2000 6 537 543 10.3109/13550280009091954 11175326

[B65] Nicolini A Ajmone-Cat MA Bernardo A Levi G Minghetti L Human immunodeficiency virus type-1 Tat protein induces nuclear factor (NF)-kappaB activation and oxidative stress in microglial cultures by independent mechanisms J Neurochem 2001 79 713 716 10.1046/j.1471-4159.2001.00568.x 11701774

[B66] Bruce-Keller AJ Barger SW Moss NI Pham JT Keller JN Nath A Pro-inflammatory and pro-oxidant properties of the HIV protein Tat in a microglial cell line: attenuation by 17 beta-estradiol J Neurochem 2001 78 1315 1324 10.1046/j.1471-4159.2001.00511.x 11579140

[B67] Bowie A O'Neill LA Oxidative stress and nuclear factor-kappaB activation: a reassessment of the evidence in the light of recent discoveries Biochem Pharmacol 2000 59 13 23 10.1016/S0006-2952(99)00296-8 10605930

[B68] Hegg CC Hu S Peterson PK Thayer SA Beta-chemokines and human immunodeficiency virus type-1 proteins evoke intracellular calcium increases in human microglia Neuroscience 2000 98 191 199 10.1016/S0306-4522(00)00101-9 10858625

[B69] Patrizio M Colucci M Levi G Human immunodeficiency virus type 1 Tat protein decreases cyclic AMP synthesis in rat microglia cultures J Neurochem 2001 77 399 407 10.1046/j.1471-4159.2001.00249.x 11299302

[B70] Aloisi F De Simone R Columba-Cabezas S Levi G Opposite effects of interferon-gamma and prostaglandin E2 on tumor necrosis factor and interleukin-10 production in microglia: a regulatory loop controlling microglia pro- and anti-inflammatory activities J Neurosci Res 1999 56 571 580 10.1002/(SICI)1097-4547(19990615)56:6<571::AID-JNR3>3.0.CO;2-P 10374812

[B71] Aloisi F Penna G Cerase J Menendez Iglesias B Adorini L IL-12 production by central nervous system microglia is inhibited by astrocytes J Immunol 1997 159 1604 1612 9257819

[B72] Caggiano AO Kraig RP Prostaglandin E receptor subtypes in cultured rat microglia and their role in reducing lipopolysaccharide-induced interleukin-1beta production J Neurochem 1999 72 565 575 10.1046/j.1471-4159.1999.0720565.x 9930728 PMC2807136

[B73] Davalos D Grutzendler J Yang G Kim JV Zuo Y Jung S Littman DR Dustin ML Gan WB ATP mediates rapid microglial response to local brain injury in vivo Nat Neurosci 2005 8 752 758 10.1038/nn1472 15895084

[B74] Minghetti L Nicolini A Polazzi E Creminon C Maclouf J Levi G Prostaglandin E2 downregulates inducible nitric oxide synthase expression in microglia by increasing cAMP levels Adv Exp Med Biol 1997 433 181 184 9561130 10.1007/978-1-4899-1810-9_37

[B75] Minghetti L Nicolini A Polazzi E Creminon C Maclouf J Levi G Inducible nitric oxide synthase expression in activated rat microglial cultures is downregulated by exogenous prostaglandin E2 and by cyclooxygenase inhibitors Glia 1997 19 152 160 10.1002/(SICI)1098-1136(199702)19:2<152::AID-GLIA6>3.0.CO;2-2 9034831

[B76] Takahashi K Prinz M Stagi M Chechneva O Neumann H TREM2-transduced myeloid precursors mediate nervous tissue debris clearance and facilitate recovery in an animal model of multiple sclerosis PLoS Med 2007 4 e124 10.1371/journal.pmed.0040124 17425404 PMC1851623

[B77] Mayr B Montminy M Transcriptional regulation by the phosphorylation-dependent factor CREB Nat Rev Mol Cell Biol 2001 2 599 609 10.1038/35085068 11483993

[B78] Kettenmann H Hoppe D Gottmann K Banati R Kreutzberg G Cultured microglial cells have a distinct pattern of membrane channels different from peritoneal macrophages J Neurosci Res 1990 26 278 287 10.1002/jnr.490260303 1697905

[B79] Korotzer AR Cotman CW Voltage-gated currents expressed by rat microglia in culture Glia 1992 6 81 88 10.1002/glia.440060202 1328052

[B80] Visentin S Agresti C Patrizio M Levi G Ion channels in rat microglia and their different sensitivity to lipopolysaccharide and interferon-gamma J Neurosci Res 1995 42 439 451 10.1002/jnr.490420402 8568930

[B81] Leone C Le Pavec G Meme W Porcheray F Samah B Dormont D Gras G Characterization of human monocyte-derived microglia-like cells Glia 2006 54 183 192 10.1002/glia.20372 16807899

[B82] Visentin S Levi G Protein kinase C involvement in the resting and interferon-gamma-induced K+ channel profile of microglial cells J Neurosci Res 1997 47 233 241 10.1002/(SICI)1097-4547(19970201)47:3<233::AID-JNR1>3.0.CO;2-J 9039645

[B83] Visentin S Renzi M Levi G Altered outward-rectifying K(+) current reveals microglial activation induced by HIV-1 Tat protein Glia 2001 33 181 190 10.1002/1098-1136(200103)33:3<181::AID-GLIA1017>3.0.CO;2-Q 11241736

[B84] Cheeseman CI Molecular mechanisms involved in the regulation of amino acid transport Prog Biophys Mol Biol 1991 55 71 84 10.1016/0079-6107(91)90001-9 1871316

[B85] Gras G Porcheray F Samah B Leone C The glutamate-glutamine cycle as an inducible, protective face of macrophage activation J Leukoc Biol 2006 80 1067 1075 10.1189/jlb.0306153 16912070

[B86] Porcheray F Leone C Samah B Rimaniol AC Dereuddre-Bosquet N Gras G Glutamate metabolism in HIV-infected macrophages: implications for the CNS Am J Physiol Cell Physiol 2006 291 C618 626 10.1152/ajpcell.00021.2006 16687472

[B87] Rimaniol AC Mialocq P Clayette P Dormont D Gras G Role of glutamate transporters in the regulation of glutathione levels in human macrophages Am J Physiol Cell Physiol 2001 281 C1964 1970 11698255 10.1152/ajpcell.2001.281.6.C1964

[B88] McArthur JC Hoover DR Bacellar H Miller EN Cohen BA Becker JT Graham NM McArthur JH Selnes OA Jacobson LP Dementia in AIDS patients: incidence and risk factors. Multicenter AIDS Cohort Study Neurology 1993 43 2245 2252 8232937 10.1212/wnl.43.11.2245

[B89] Emerman M HIV-1, Vpr and the cell cycle Curr Biol 1996 6 1096 1103 10.1016/S0960-9822(02)00676-0 8805364

[B90] Dragic T Litwin V Allaway GP Martin SR Huang Y Nagashima KA Cayanan C Maddon PJ Koup RA Moore JP Paxton WA HIV-1 entry into CD4+ cells is mediated by the chemokine receptor CC-CKR-5 Nature 1996 381 667 673 10.1038/381667a0 8649512

[B91] Subbramanian RA Kessous-Elbaz A Lodge R Forget J Yao XJ Bergeron D Cohen EA Human immunodeficiency virus type 1 Vpr is a positive regulator of viral transcription and infectivity in primary human macrophages J Exp Med 1998 187 1103 1111 10.1084/jem.187.7.1103 9529326 PMC2212198

[B92] Vodicka MA Koepp DM Silver PA Emerman M HIV-1 Vpr interacts with the nuclear transport pathway to promote macrophage infection Genes Dev 1998 12 175 185 10.1101/gad.12.2.175 9436978 PMC316441

[B93] Jacquot G Le Rouzic E David A Mazzolini J Bouchet J Bouaziz S Niedergang F Pancino G Benichou S Localization of HIV-1 Vpr to the nuclear envelope: impact on Vpr functions and virus replication in macrophages Retrovirology 2007 4 84 10.1186/1742-4690-4-84 18039376 PMC2211753

[B94] Heinzinger NK Bukinsky MI Haggerty SA Ragland AM Kewalramani V Lee MA Gendelman HE Ratner L Stevenson M Emerman M The Vpr protein of human immunodeficiency virus type 1 influences nuclear localization of viral nucleic acids in nondividing host cells Proc Natl Acad Sci USA 1994 91 7311 7315 10.1073/pnas.91.15.7311 8041786 PMC44389

[B95] Ogawa K Shibata R Kiyomasu T Higuchi I Kishida Y Ishimoto A Adachi A Mutational analysis of the human immunodeficiency virus vpr open reading frame J Virol 1989 63 4110 4114 2474678 10.1128/jvi.63.9.4110-4114.1989 PMC251018

[B96] Westervelt P Henkel T Trowbridge DB Orenstein J Heuser J Gendelman HE Ratner L Dual regulation of silent and productive infection in monocytes by distinct human immunodeficiency virus type 1 determinants J Virol 1992 66 3925 3931 1533883 10.1128/jvi.66.6.3925-3931.1992 PMC241183

[B97] Balotta C Lusso P Crowley R Gallo RC Franchini G Antisense phosphorothioate oligodeoxynucleotides targeted to the vpr gene inhibit human immunodeficiency virus type 1 replication in primary human macrophages J Virol 1993 67 4409 4414 8510229 10.1128/jvi.67.7.4409-4414.1993 PMC237816

[B98] Balliet JW Kolson DL Eiger G Kim FM McGann KA Srinivasan A Collman R Distinct effects in primary macrophages and lymphocytes of the human immunodeficiency virus type 1 accessory genes vpr, vpu, and nef: mutational analysis of a primary HIV-1 isolate Virology 1994 200 623 631 10.1006/viro.1994.1225 8178448

[B99] Albright AV Shieh JT Itoh T Lee B Pleasure D O'Connor MJ Doms RW Gonzalez-Scarano F Microglia express CCR5, CXCR4, and CCR3, but of these, CCR5 is the principal coreceptor for human immunodeficiency virus type 1 dementia isolates J Virol 1999 73 205 213 9847323 10.1128/jvi.73.1.205-213.1999 PMC103824

[B100] Emerman M Malim MH HIV-1 regulatory/accessory genes: keys to unraveling viral and host cell biology Science 1998 280 1880 1884 10.1126/science.280.5371.1880 9632380

[B101] Frankel AD Young JA HIV-1: fifteen proteins and an RNA Annu Rev Biochem 1998 67 1 25 10.1146/annurev.biochem.67.1.1 9759480

[B102] Bukrinsky M Adzhubei A Viral protein R of HIV-1 Rev Med Virol 1999 9 39 49 10.1002/(SICI)1099-1654(199901/03)9:1<39::AID-RMV235>3.0.CO;2-3 10371671

[B103] Stewart SA Poon B Song JY Chen IS Human immunodeficiency virus type 1 vpr induces apoptosis through caspase activation J Virol 2000 74 3105 3111 10.1128/JVI.74.7.3105-3111.2000 10708425 PMC111809

[B104] He J Choe S Walker R Di Marzio P Morgan DO Landau NR Human immunodeficiency virus type 1 viral protein R (Vpr) arrests cells in the G2 phase of the cell cycle by inhibiting p34cdc2 activity J Virol 1995 69 6705 6711 7474080 10.1128/jvi.69.11.6705-6711.1995 PMC189580

[B105] Jowett JB Planelles V Poon B Shah NP Chen ML Chen IS The human immunodeficiency virus type 1 vpr gene arrests infected T cells in the G2 + M phase of the cell cycle J Virol 1995 69 6304 6313 7666531 10.1128/jvi.69.10.6304-6313.1995 PMC189529

[B106] Re F Braaten D Franke EK Luban J Human immunodeficiency virus type 1 Vpr arrests the cell cycle in G2 by inhibiting the activation of p34cdc2-cyclin B J Virol 1995 69 6859 6864 7474100 10.1128/jvi.69.11.6859-6864.1995 PMC189600

[B107] Sawaya BE Khalili K Mercer WE Denisova L Amini S Cooperative actions of HIV-1 Vpr and p53 modulate viral gene transcription J Biol Chem 1998 273 20052 20057 10.1074/jbc.273.32.20052 9685344

[B108] Bartz SR Rogel ME Emerman M Human immunodeficiency virus type 1 cell cycle control: Vpr is cytostatic and mediates G2 accumulation by a mechanism which differs from DNA damage checkpoint control J Virol 1996 70 2324 2331 8642659 10.1128/jvi.70.4.2324-2331.1996 PMC190074

[B109] Yao XJ Mouland AJ Subbramanian RA Forget J Rougeau N Bergeron D Cohen EA Vpr stimulates viral expression and induces cell killing in human immunodeficiency virus type 1-infected dividing Jurkat T cells J Virol 1998 72 4686 4693 9573232 10.1128/jvi.72.6.4686-4693.1998 PMC109992

[B110] Watanabe N Yamaguchi T Akimoto Y Rattner JB Hirano H Nakauchi H Induction of M-phase arrest and apoptosis after HIV-1 Vpr expression through uncoupling of nuclear and centrosomal cycle in HeLa cells Exp Cell Res 2000 258 261 269 10.1006/excr.2000.4908 10896777

[B111] Jacotot E Ferri KF El Hamel C Brenner C Druillennec S Hoebeke J Rustin P Metivier D Lenoir C Geuskens M Vieira HL Loeffler M Belzacq AS Briand JP Zamzami N Edelman L Xie ZH Reed JC Roques BP Kroemer G Control of mitochondrial membrane permeabilization by adenine nucleotide translocator interacting with HIV-1 viral protein rR and Bcl-2 J Exp Med 2001 193 509 519 10.1084/jem.193.4.509 11181702 PMC2195906

[B112] Gummuluru S Emerman M Cell cycle- and Vpr-mediated regulation of human immunodeficiency virus type 1 expression in primary and transformed T-cell lines J Virol 1999 73 5422 5430 10364289 10.1128/jvi.73.7.5422-5430.1999 PMC112598

[B113] Wang L Mukherjee S Jia F Narayan O Zhao LJ Interaction of virion protein Vpr of human immunodeficiency virus type 1 with cellular transcription factor Sp1 and trans-activation of viral long terminal repeat J Biol Chem 1995 270 25564 25569 10.1074/jbc.270.43.25564 7592727

[B114] Sawaya BE Khalili K Gordon J Taube R Amini S Cooperative interaction between HIV-1 regulatory proteins Tat and Vpr modulates transcription of the viral genome J Biol Chem 2000 275 35209 35214 10.1074/jbc.M005197200 10931842

[B115] Varin A Decrion AZ Sabbah E Quivy V Sire J Van Lint C Roques BP Aggarwal BB Herbein G Synthetic Vpr protein activates activator protein-1, c-Jun N-terminal kinase, and NF-kappaB and stimulates HIV-1 transcription in promonocytic cells and primary macrophages J Biol Chem 2005 280 42557 42567 10.1074/jbc.M502211200 16243842

[B116] Eckstein DA Sherman MP Penn ML Chin PS De Noronha CM Greene WC Goldsmith MA HIV-1 Vpr enhances viral burden by facilitating infection of tissue macrophages but not nondividing CD4+ T cells J Exp Med 2001 194 1407 1419 10.1084/jem.194.10.1407 11714748 PMC2193684

[B117] Vazquez N Greenwell-Wild T Marinos NJ Swaim WD Nares S Ott DE Schubert U Henklein P Orenstein JM Sporn MB Wahl SM Human immunodeficiency virus type 1-induced macrophage gene expression includes the p21 gene, a target for viral regulation J Virol 2005 79 4479 4491 10.1128/JVI.79.7.4479-4491.2005 15767448 PMC1061522

[B118] Muthumani K Hwang DS Choo AY Mayilvahanan S Dayes NS Thieu KP Weiner DB HIV-1 Vpr inhibits the maturation and activation of macrophages and dendritic cells in vitro Int Immunol 2005 17 103 116 10.1093/intimm/dxh190 15611322

[B119] Muthumani K Kudchodkar S Papasavvas E Montaner LJ Weiner DB Ayyavoo V HIV-1 Vpr regulates expression of beta chemokines in human primary lymphocytes and macrophages J Leukoc Biol 2000 68 366 372 10985253

[B120] Muthumani K Hwang DS Dayes NS Kim JJ Weiner DB The HIV-1 accessory gene vpr can inhibit antigen-specific immune function DNA Cell Biol 2002 21 689 695 10.1089/104454902760330237 12396612

[B121] Herbein G Keshav S Collin M Montaner LJ Gordon S HIV-1 induces tumour necrosis factor and IL-1 gene expression in primary human macrophages independent of productive infection Clin Exp Immunol 1994 95 442 449 7511077 10.1111/j.1365-2249.1994.tb07016.x PMC1535095

[B122] Cocchi F DeVico AL Garzino-Demo A Arya SK Gallo RC Lusso P Identification of RANTES, MIP-1 alpha, and MIP-1 beta as the major HIV-suppressive factors produced by CD8+ T cells Science 1995 270 1811 1815 10.1126/science.270.5243.1811 8525373

[B123] Alkhatib G Combadiere C Broder CC Feng Y Kennedy PE Murphy PM Berger EA CC CKR5: a RANTES, MIP-1alpha, MIP-1beta receptor as a fusion cofactor for macrophage-tropic HIV-1 Science 1996 272 1955 1958 10.1126/science.272.5270.1955 8658171

[B124] Choe H Farzan M Sun Y Sullivan N Rollins B Ponath PD Wu L Mackay CR LaRosa G Newman W Gerard N Gerard C Sodroski J The beta-chemokine receptors CCR3 and CCR5 facilitate infection by primary HIV-1 isolates Cell 1996 85 1135 1148 10.1016/S0092-8674(00)81313-6 8674119

[B125] Deng H Liu R Ellmeier W Choe S Unutmaz D Burkhart M Di Marzio P Marmon S Sutton RE Hill CM Davis CB Peiper SC Schall TJ Littman DR Landau NR Identification of a major co-receptor for primary isolates of HIV-1 Nature 1996 381 661 666 10.1038/381661a0 8649511

[B126] Liu Y Hao W Letiembre M Walter S Kulanga M Neumann H Fassbender K Suppression of microglial inflammatory activity by myelin phagocytosis: role of p47-PHOX-mediated generation of reactive oxygen species J Neurosci 2006 26 12904 12913 10.1523/JNEUROSCI.2531-06.2006 17167081 PMC6674962

[B127] Doranz BJ Rucker J Yi Y Smyth RJ Samson M Peiper SC Parmentier M Collman RG Doms RW A dual-tropic primary HIV-1 isolate that uses fusin and the beta-chemokine receptors CKR-5, CKR-3, and CKR-2b as fusion cofactors Cell 1996 85 1149 1158 10.1016/S0092-8674(00)81314-8 8674120

[B128] Schmidtmayerova H Sherry B Bukrinsky M Chemokines and HIV replication Nature 1996 382 767 10.1038/382767a0 8752270

[B129] Wang J Roderiquez G Oravecz T Norcross MA Cytokine regulation of human immunodeficiency virus type 1 entry and replication in human monocytes/macrophages through modulation of CCR5 expression J Virol 1998 72 7642 7647 9696868 10.1128/jvi.72.9.7642-7647.1998 PMC110028

[B130] Verani A Gras G Pancino G Macrophages and HIV-1: dangerous liaisons Mol Immunol 2005 42 195 212 10.1016/j.molimm.2004.06.020 15488608

[B131] Melikyan GB Common principles and intermediates of viral protein-mediated fusion: the HIV-1 paradigm Retrovirology 2008 5 111 10.1186/1742-4690-5-111 19077194 PMC2633019

[B132] Bergamini A Faggioli E Bolacchi F Gessani S Cappannoli L Uccella I Demin F Capozzi M Cicconi R Placido R Vendetti S Colizzi GM Rocchi G Enhanced production of tumor necrosis factor-alpha and interleukin-6 due to prolonged response to lipopolysaccharide in human macrophages infected in vitro with human immunodeficiency virus type 1 J Infect Dis 1999 179 832 842 10.1086/314662 10068578

[B133] Clouse KA Cosentino LM Weih KA Pyle SW Robbins PB Hochstein HD Natarajan V Farrar WL The HIV-1 gp120 envelope protein has the intrinsic capacity to stimulate monokine secretion J Immunol 1991 147 2892 2901 1918997

[B134] Karsten V Gordon S Kirn A Herbein G HIV-1 envelope glycoprotein gp120 down-regulates CD4 expression in primary human macrophages through induction of endogenous tumour necrosis factor-alpha Immunology 1996 88 55 60 10.1046/j.1365-2567.1996.d01-648.x 8707350 PMC1456460

[B135] Merrill JE Koyanagi Y Chen IS Interleukin-1 and tumor necrosis factor alpha can be induced from mononuclear phagocytes by human immunodeficiency virus type 1 binding to the CD4 receptor J Virol 1989 63 4404 4408 2789293 10.1128/jvi.63.10.4404-4408.1989 PMC251058

[B136] Choe W Volsky DJ Potash MJ Induction of rapid and extensive beta-chemokine synthesis in macrophages by human immunodeficiency virus type 1 and gp120, independently of their coreceptor phenotype J Virol 2001 75 10738 10745 10.1128/JVI.75.22.10738-10745.2001 11602715 PMC114655

[B137] Bailer RT Lee B Montaner LJ IL-13 and TNF-alpha inhibit dual-tropic HIV-1 in primary macrophages by reduction of surface expression of CD4, chemokine receptors CCR5, CXCR4 and post-entry viral gene expression Eur J Immunol 2000 30 1340 1349 10.1002/(SICI)1521-4141(200005)30:5<1340::AID-IMMU1340>3.0.CO;2-L 10820380

[B138] Faltynek CR Finch LR Miller P Overton WR Treatment with recombinant IFN-gamma decreases cell surface CD4 levels on peripheral blood monocytes and on myelomonocyte cell lines J Immunol 1989 142 500 508 2492048

[B139] Montaner LJ Herbein G Gordon S Regulation of macrophage activation and HIV replication Adv Exp Med Biol 1995 374 47 56 7572400 10.1007/978-1-4615-1995-9_5

[B140] Cotter RL Zheng J Che M Niemann D Liu Y He J Thomas E Gendelman HE Regulation of human immunodeficiency virus type 1 infection, beta-chemokine production, and CCR5 expression in CD40L-stimulated macrophages: immune control of viral entry J Virol 2001 75 4308 4320 10.1128/JVI.75.9.4308-4320.2001 11287580 PMC114176

[B141] Herbein G Montaner LJ Gordon S Tumor necrosis factor alpha inhibits entry of human immunodeficiency virus type 1 into primary human macrophages: a selective role for the 75-kilodalton receptor J Virol 1996 70 7388 7397 8892857 10.1128/jvi.70.11.7388-7397.1996 PMC190806

[B142] Herbein G Gordon S 55- and 75-kilodalton tumor necrosis factor receptors mediate distinct actions in regard to human immunodeficiency virus type 1 replication in primary human macrophages J Virol 1997 71 4150 4156 9094699 10.1128/jvi.71.5.4150-4156.1997 PMC191574

[B143] Meylan PR Guatelli JC Munis JR Richman DD Kornbluth RS Mechanisms for the inhibition of HIV replication by interferons-alpha, -beta, and -gamma in primary human macrophages Virology 1993 193 138 148 10.1006/viro.1993.1110 7679856

[B144] Zaitseva M Lee S Lapham C Taffs R King L Romantseva T Manischewitz J Golding H Interferon gamma and interleukin 6 modulate the susceptibility of macrophages to human immunodeficiency virus type 1 infection Blood 2000 96 3109 3117 11049991

[B145] Cremer I Vieillard V De Maeyer E Retrovirally mediated IFN-beta transduction of macrophages induces resistance to HIV, correlated with up-regulation of RANTES production and down-regulation of C-C chemokine receptor-5 expression J Immunol 2000 164 1582 1587 10640778 10.4049/jimmunol.164.3.1582

[B146] Hewson TJ Logie JJ Simmonds P Howie SE A CCR5-dependent novel mechanism for type 1 HIV gp120 induced loss of macrophage cell surface CD4 J Immunol 2001 166 4835 4842 11290759 10.4049/jimmunol.166.8.4835

[B147] Lane BR Markovitz DM Woodford NL Rochford R Strieter RM Coffey MJ TNF-alpha inhibits HIV-1 replication in peripheral blood monocytes and alveolar macrophages by inducing the production of RANTES and decreasing C-C chemokine receptor 5 (CCR5) expression J Immunol 1999 163 3653 3661 10490959

[B148] Coffey MJ Woffendin C Phare SM Strieter RM Markovitz DM RANTES inhibits HIV-1 replication in human peripheral blood monocytes and alveolar macrophages Am J Physiol 1997 272 L1025 1029 9176270 10.1152/ajplung.1997.272.5.L1025

[B149] Jiang Y Jolly PE Effect of beta-chemokines on human immunodeficiency virus type 1 replication, binding, uncoating, and CCR5 receptor expression in human monocyte-derived macrophages J Hum Virol 1999 2 123 132 10413363

[B150] Capobianchi MR Abbate I Antonelli G Turriziani O Dolei A Dianzani F Inhibition of HIV type 1 BaL replication by MIP-1alpha, MIP-1beta, and RANTES in macrophages AIDS Res Hum Retroviruses 1998 14 233 240 10.1089/aid.1998.14.233 9491913

[B151] Ylisastigui L Amzazi S Bakri Y Vizzavona J Vita C Gluckman JC Benjouad A Effect of RANTES on the infection of monocyte-derived primary macrophages by human immunodeficiency virus type 1 and type 2 Biomedicine & Pharmacotherapy 1998 52 447 453 10.1016/s0753-3322(99)80023-7 9921414

[B152] Stantchev TS Broder CC Consistent and significant inhibition of human immunodeficiency virus type 1 envelope-mediated membrane fusion by beta-chemokines (RANTES) in primary human macrophages J Infect Dis 2000 182 68 78 10.1086/315700 10882583

[B153] Moriuchi H Moriuchi M Fauci AS Nuclear factor-kappa B potently up-regulates the promoter activity of RANTES, a chemokine that blocks HIV infection J Immunol 1997 158 3483 3491 9120310

[B154] Wahl LM Corcoran ML Pyle SW Arthur LO Harel-Bellan A Farrar WL Human immunodeficiency virus glycoprotein (gp120) induction of monocyte arachidonic acid metabolites and interleukin 1 Proc Natl Acad Sci USA 1989 86 621 625 10.1073/pnas.86.2.621 2536171 PMC286524

[B155] Klasse PJ Moore JP Is there enough gp120 in the body fluids of HIV-1-infected individuals to have biologically significant effects? Virology 2004 323 1 8 15165814 10.1016/j.virol.2004.03.003

[B156] Yi Y Lee C Liu QH Freedman BD Collman RG Chemokine receptor utilization and macrophage signaling by human immunodeficiency virus type 1 gp120: Implications for neuropathogenesis J Neurovirol 2004 10 Suppl 1 91 96 14982745 10.1080/753312758

[B157] Liu QH Williams DA McManus C Baribaud F Doms RW Schols D De Clercq E Kotlikoff MI Collman RG Freedman BD HIV-1 gp120 and chemokines activate ion channels in primary macrophages through CCR5 and CXCR4 stimulation Proc Natl Acad Sci USA 2000 97 4832 4837 10.1073/pnas.090521697 10758170 PMC18318

[B158] Herbein G Mahlknecht U Batliwalla F Gregersen P Pappas T Butler J O'Brien WA Verdin E Apoptosis of CD8+ T cells is mediated by macrophages through interaction of HIV gp120 with chemokine receptor CXCR4 Nature 1998 395 189 194 10.1038/26026 9744279

[B159] Decrion AZ Varin A Estavoyer JM Herbein G CXCR4-Mediated T Cell Apoptosis in HIV infection Journal of General Virology 2004 85 1471 8 10.1099/vir.0.79933-0 15166430

[B160] Kenakin T Ligand-selective receptor conformations revisited: the promise and the problem Trends Pharmacol Sci 2003 24 346 354 10.1016/S0165-6147(03)00167-6 12871667

[B161] Del Corno M Liu QH Schols D de Clercq E Gessani S Freedman BD Collman RG HIV-1 gp120 and chemokine activation of Pyk2 and mitogen-activated protein kinases in primary macrophages mediated by calcium-dependent, pertussis toxin-insensitive chemokine receptor signaling Blood 2001 98 2909 2916 10.1182/blood.V98.10.2909 11698270

[B162] Tomkowicz B Lee C Ravyn V Cheung R Ptasznik A Collman RG The Src kinase Lyn is required for CCR5 signaling in response to MIP-1beta and R5 HIV-1 gp120 in human macrophages Blood 2006 108 1145 1150 10.1182/blood-2005-12-012815 16621960 PMC1895866

[B163] Lynch GW Turville S Carter B Sloane AJ Chan A Muljadi N Li S Low L Armati P Raison R Zoellner H Williamson P Cunningham A Church WB Marked differences in the structures and protein associations of lymphocyte and monocyte CD4: resolution of a novel CD4 isoform Immunol Cell Biol 2006 84 154 165 10.1111/j.1440-1711.2005.01403.x 16519733

[B164] Chugh P Bradel-Tretheway B Monteiro-Filho CM Planelles V Maggirwar SB Dewhurst S Kim B Akt inhibitors as an HIV-1 infected macrophage-specific anti-viral therapy Retrovirology 2008 5 11 10.1186/1742-4690-5-11 18237430 PMC2265748

[B165] Benkirane M Jeang KT Devaux C The cytoplasmic domain of CD4 plays a critical role during the early stages of HIV infection in T-cells Embo J 1994 13 5559 5569 7988553 10.1002/j.1460-2075.1994.tb06893.x PMC395519

[B166] Briant L Coudronniere N Robert-Hebmann V Benkirane M Devaux C Binding of HIV-1 virions or gp120-anti-gp120 immune complexes to HIV-1-infected quiescent peripheral blood mononuclear cells reveals latent infection J Immunol 1996 156 3994 4004 8621941

[B167] Briant L Robert-Hebmann V Acquaviva C Pelchen-Matthews A Marsh M Devaux C The protein tyrosine kinase p56lck is required for triggering NF-kappaB activation upon interaction of human immunodeficiency virus type 1 envelope glycoprotein gp120 with cell surface CD4 J Virol 1998 72 6207 6214 9621091 10.1128/jvi.72.7.6207-6214.1998 PMC110439

[B168] Chirmule N Kalyanaraman VS Pahwa S Signals transduced through the CD4 molecule on T lymphocytes activate NF-kappa B Biochem Biophys Res Commun 1994 203 498 505 10.1006/bbrc.1994.2210 7915519

[B169] Popik W Pitha PM Binding of human immunodeficiency virus type 1 to CD4 induces association of Lck and Raf-1 and activates Raf-1 by a Ras-independent pathway Mol Cell Biol 1996 16 6532 6541 8887682 10.1128/mcb.16.11.6532 PMC231655

[B170] Popik W Hesselgesser JE Pitha PM Binding of human immunodeficiency virus type 1 to CD4 and CXCR4 receptors differentially regulates expression of inflammatory genes and activates the MEK/ERK signaling pathway J Virol 1998 72 6406 6413 9658081 10.1128/jvi.72.8.6406-6413.1998 PMC109793

[B171] Schmid-Antomarchi H Benkirane M Breittmayer V Husson H Ticchioni M Devaux C Rossi B HIV induces activation of phosphatidylinositol 4-kinase and mitogen-activated protein kinase by interacting with T cell CD4 surface molecules Eur J Immunol 1996 26 717 720 10.1002/eji.1830260331 8605943

[B172] Biard-Piechaczyk M Robert-Hebmann V Richard V Roland J Hipskind RA Devaux C Caspase-dependent apoptosis of cells expressing the chemokine receptor CXCR4 is induced by cell membrane-associated human immunodeficiency virus type 1 envelope glycoprotein (gp120) Virology 2000 268 329 344 10.1006/viro.1999.0151 10704341

[B173] Duong LT Rodan GA PYK2 is an adhesion kinase in macrophages, localized in podosomes and activated by beta(2)-integrin ligation Cell Motil Cytoskeleton 2000 47 174 188 10.1002/1097-0169(200011)47:3<174::AID-CM2>3.0.CO;2-N 11056520

[B174] Davis CB Dikic I Unutmaz D Hill CM Arthos J Siani MA Thompson DA Schlessinger J Littman DR Signal transduction due to HIV-1 envelope interactions with chemokine receptors CXCR4 or CCR5 J Exp Med 1997 186 1793 1798 10.1084/jem.186.10.1793 9362541 PMC2199136

[B175] Okigaki M Davis C Falasca M Harroch S Felsenfeld DP Sheetz MP Schlessinger J Pyk2 regulates multiple signaling events crucial for macrophage morphology and migration Proc Natl Acad Sci USA 2003 100 10740 10745 10.1073/pnas.1834348100 12960403 PMC196873

[B176] Herbein G Coaquette A Perez-Bercoff D Pancino G Macrophage activation and HIV infection: can the Trojan horse turn into a fortress? Curr Mol Med 2002 2 723 738 10.2174/1566524023361844 12462393

[B177] Finkel TH Tudor-Williams G Banda NK Cotton MF Curiel T Monks C Baba TW Ruprecht RM Kupfer A Apoptosis occurs predominantly in bystander cells and not in productively infected cells of HIV- and SIV-infected lymph nodes Nat Med 1995 1 129 134 10.1038/nm0295-129 7585008

[B178] Katsikis PD Garcia-Ojeda ME Torres-Roca JF Greenwald DR Herzenberg LA HIV type 1 Tat protein enhances activation-but not Fas (CD95)-induced peripheral blood T cell apoptosis in healthy individuals Int Immunol 1997 9 835 841 10.1093/intimm/9.6.835 9199966

[B179] Groux H Torpier G Monte D Mouton Y Capron A Ameisen JC Activation-induced death by apoptosis in CD4+ T cells from human immunodeficiency virus-infected asymptomatic individuals J Exp Med 1992 175 331 340 10.1084/jem.175.2.331 1346269 PMC2119133

[B180] Oyaizu N McCloskey TW Coronesi M Chirmule N Kalyanaraman VS Pahwa S Accelerated apoptosis in peripheral blood mononuclear cells (PBMCs) from human immunodeficiency virus type-1 infected patients and in CD4 cross-linked PBMCs from normal individuals Blood 1993 82 3392 3400 7902137

[B181] Geleziunas R Xu W Takeda K Ichijo H Greene WC HIV-1 Nef inhibits ASK1-dependent death signalling providing a potential mechanism for protecting the infected host cell Nature 2001 410 834 838 10.1038/35071111 11298454

[B182] Yoon K Jeong JG Kim S Stable expression of human immunodeficiency virus type 1 Nef confers resistance against Fas-mediated apoptosis AIDS Res Hum Retroviruses 2001 17 99 104 10.1089/08892220150217184 11177389

[B183] Mahlknecht U Herbein G Macrophages and T-cell apoptosis in HIV infection: a leading role for accessory cells? Trends Immunol 2001 22 256 260 10.1016/S1471-4906(01)01898-1 11323283

[B184] Mahlknecht U Deng C Lu MC Greenough TC Sullivan JL O'Brien WA Herbein G Resistance to apoptosis in HIV-infected CD4+ T lymphocytes is mediated by macrophages: role for Nef and immune activation in viral persistence J Immunol 2000 165 6437 6446 11086083 10.4049/jimmunol.165.11.6437

[B185] Badley AD McElhinny JA Leibson PJ Lynch DH Alderson MR Paya CV Upregulation of Fas ligand expression by human immunodeficiency virus in human macrophages mediates apoptosis of uninfected T lymphocytes J Virol 1996 70 199 206 8523526 10.1128/jvi.70.1.199-206.1996 PMC189805

[B186] Badley AD Dockrell D Simpson M Schut R Lynch DH Leibson P Paya CV Macrophage-dependent apoptosis of CD4+ T lymphocytes from HIV-infected individuals is mediated by FasL and tumor necrosis factor J Exp Med 1997 185 55 64 10.1084/jem.185.1.55 8996241 PMC2196110

[B187] Gendelman HE Orenstein JM Baca LM Weiser B Burger H Kalter DC Meltzer MS The macrophage in the persistence and pathogenesis of HIV infection Aids 1989 3 475 495 2508709 10.1097/00002030-198908000-00001

[B188] Zheng L Fisher G Miller RE Peschon J Lynch DH Lenardo MJ Induction of apoptosis in mature T cells by tumour necrosis factor Nature 1995 377 348 351 10.1038/377348a0 7566090

[B189] Grell M Douni E Wajant H Lohden M Clauss M Maxeiner B Georgopoulos S Lesslauer W Kollias G Pfizenmaier K The transmembrane form of tumor necrosis factor is the prime activating ligand of the 80 kDa tumor necrosis factor receptor Cell 1995 83 793 802 10.1016/0092-8674(95)90192-2 8521496

[B190] Lahdevirta J Maury CP Teppo AM Repo H Elevated levels of circulating cachectin/tumor necrosis factor in patients with acquired immunodeficiency syndrome Am J Med 1988 85 289 291 10.1016/0002-9343(88)90576-1 3414726

[B191] Poonia B Pauza CD Salvato MS Role of the Fas/FasL pathway in HIV or SIV disease Retrovirology 2009 6 91 10.1186/1742-4690-6-91 19832988 PMC2772842

[B192] Galea I Palin K Newman TA Van Rooijen N Perry VH Boche D Mannose receptor expression specifically reveals perivascular macrophages in normal, injured, and diseased mouse brain Glia 2005 49 375 384 10.1002/glia.20124 15538754

[B193] Blair PJ Boise LH Perfetto SP Levine BL McCrary G Wagner KF St Louis DC Thompson CB Siegel JN June CH Impaired induction of the apoptosis-protective protein Bcl-xL in activated PBMC from asymptomatic HIV-infected individuals J Clin Immunol 1997 17 234 246 10.1023/A:1027310612323 9168404

[B194] Kaul M Lipton SA Chemokines and activated macrophages in HIV gp120-induced neuronal apoptosis Proc Natl Acad Sci USA 1999 96 8212 8216 10.1073/pnas.96.14.8212 10393974 PMC22214

[B195] Wu DT Woodman SE Weiss JM McManus CM D'Aversa TG Hesselgesser J Major EO Nath A Berman JW Mechanisms of leukocyte trafficking into the CNS J Neurovirol 2000 6 Suppl 1 S82 85 10871769

[B196] Swingler S Mann A Jacque J Brichacek B Sasseville VG Williams K Lackner AA Janoff EN Wang R Fisher D Stevenson M HIV-1 Nef mediates lymphocyte chemotaxis and activation by infected macrophages Nat Med 1999 5 997 103 10.1038/12433 10470075 PMC9513713

[B197] Smit TK Wang B Ng T Osborne R Brew B Saksena NK Varied tropism of HIV-1 isolates derived from different regions of adult brain cortex discriminate between patients with and without AIDS dementia complex (ADC): evidence for neurotropic HIV variants Virology 2001 279 509 526 10.1006/viro.2000.0681 11162807

[B198] Duh EJ Maury WJ Folks TM Fauci AS Rabson AB Tumor necrosis factor alpha activates human immunodeficiency virus type 1 through induction of nuclear factor binding to the NF-kappa B sites in the long terminal repeat Proc Natl Acad Sci USA 1989 86 5974 5978 10.1073/pnas.86.15.5974 2762307 PMC297754

[B199] Antoni BA Sabbatini P Rabson AB White E Inhibition of apoptosis in human immunodeficiency virus-infected cells enhances virus production and facilitates persistent infection J Virol 1995 69 2384 2392 7884884 10.1128/jvi.69.4.2384-2392.1995 PMC188911

